# Global Health Action at 15 – revisiting its rationale

**DOI:** 10.1080/16549716.2021.1965863

**Published:** 2021-09-09

**Authors:** Stig Wall, Maria Emmelin, Ingela Krantz, Maria Nilsson, Fredrik Norström, Julia Schröders, Jennifer Stewart Williams, Per-Olof Östergren

**Affiliations:** Department of Epidemiology and Global Health, Umeå University, Umeå, Sweden; Department of Social Medicine and Global Health, Lund University, Lund, Sweden

*Global Health Action* was launched almost 15 years ago in a world of increasing global interdependence. The Journal’s name and motivation resonates with Robert Beaglehole’s esteemed definition: ‘global health is a collaborative trans-national research and action for promoting health for all’ [1]. Our mission was phrased ‘to address critical issues in global health and facilitate collaboration between and within the North and the South’ [2].

Globalization poses threats to environmental sustainability as well as to economic and social development, especially in low- and middle-income countries. Global health researchers, policy makers and practitioners are concerned with how global processes affect health and wellbeing. They share a commitment to reducing the drivers of unfair inequalities in health and access to care within and between populations. These differences also manifest in research capacity and access to evidence and health information.

Two notable developments provided the impetus for the launch of *Global Health Action*. One was the new demands placed on public health research and policy by globalization. The other was the impact of the internet on scientific publishing with changing business models moving scientific publishing towards Open Access.

Globalization has inspired greater research collaboration and led to the proliferation of scientific fora for discussion and dissemination. But the benefits accrue disproportionately to wealthier countries with embedded research cultures and greater access to research outputs. This is evident in Europe and North America – the so-called ‘North’. The contrasts are strong for medical and health research whereby the world’s poorest countries in the ‘South’ have the highest burdens of morbidity and mortality but are also underrepresented in scientific collaborations and publications. These deficits impede the development of contextually relevant public health policies and programs by governments to address critical health, social, economic and environmental issues in their populations.

Global health requires a multi-disciplinary workforce with a broad vision of public health, and an ability to work collaboratively across disciplines, sectors and cultures. Training, upskilling, capacity building and knowledge sharing activities are essential for effective research and policy development. Globalization challenges are to be viewed through a ‘global lens’ embracing diversity and inclusion. *Global Health Action* was conceived and founded in response to these challenges.

*Global Health Action* was launched at the start of an internet-based publishing transition. Yesterday´s metaphor was that ‘knowledge’ was perceived as a published article or product – a property created by researchers but owned by publishers and archived by libraries. This contrasts starkly with today´s metaphor regarding knowledge as a ‘common good’. Twenty years ago, researchers supported the traditional model of reading and writing articles for submission to hard copy journals which were accessible only through institutional subscriptions. This status quo was reinforced by the interests of publishing houses, universities, libraries and researchers in the global North. The consequences incorporated a ‘westernized’ outlook with limited collaboration and marginal global outreach beyond institutional research portfolios.

Some researchers, curious about what the world looked like outside their own reality and how people in what was then called ‘developing countries’ lived, executed ‘parachute’ investigations to harvest data for publication. This practice highlighted two critical issues. First, the research process was rarely inclusive of local researchers as authors thereby missing opportunities for building locally driven research cultures. Second, the coverage of low- and middle-income countries in medical and health journals was unsystematic and episodic, not reflecting their interwoven burden of morbidity, mortality and poverty.

*Global Health Action* was one of the first journals in medicine and health to adopt open access publishing. This was thanks to a 50/50 ownership agreement and collaboration with an innovative, female-governed publishing house, *Co-Action Publishing*. At the time *Co-Action* was one of few publishers offering a genuine open access model. Underpinning this partnership was the mutual commitment that open access contributes directly to the democratization of knowledge and as such, can help reduce the digital divide between rich and poor countries.

In 2006, prior to the launch of *Global Health Action*, an estimated 1,346,000 scientific articles were published in 23,750 peer-reviewed journals. In 2004, King reported in *Nature* that researchers in eight leading industrialized countries produce almost 85% of the world’s most cited publications, while 163 other countries, mostly developing nations, account for less than 2.5% [3]. Around the same time a survey in *BMJ* found that, of 2,384 articles published in six leading tropical medicine journals in 2000–02, 76% lacked co-authors from low-income countries and only five percent of the articles published in the six leading tropical medicine journals in 2000–02 were generated exclusively by scientists from these countries [4]. An ongoing bibliometric study which looks at published health research in Somalia found that 69% of papers published during the past ten years had exclusively non-Somali authors, and only six percent had a Somali affiliated first author (unpublished data, 2021). These numbers expose the endemic practices of scientific colonialism and ‘safari research’ that widely underpin much academic publishing.

## Some milestones during the first 15 years

In our inaugural editorial [2] we shared a vision to address the widening gap between the winners and losers of globalization and the ‘know-do’ gap. This is achievable by fueling a hands-on approach to global health challenges. Our point of departure was that open access would facilitate rapid dissemination and equal access to information for both rich and poor. We particularly welcomed manuscripts from low- and middle-income countries, while also encouraging publications from so called South-South and South-North collaborations. Fostering such collaborations is also a key issue in the Agenda 2030.

All papers published in *Global Health Action* were, and still are, expected to address a global agenda and include a strong policy or implementation component. Authors could, for example, emphasize the foundations of health research (health information), underlying epidemiological causes (health determinants), actions for health and their effects (health interventions), the impact of the global physical and biological changes (environmental change and health) and/or the roles of health care and the importance of gender perspectives (health systems and gender).

During the first five years we focused on four areas which differentiate *Global Health Action* from many other journals in the same field. These are: *mentorship* for young researchers from low- and middle-income countries; *PhD Review articles* to help emerging researchers kickstart and sustain their research careers; *Study Design* articles describing research protocols possibly involving long term collaborations, and *Capacity Building* articles documenting research and training activities in low- and middle-income countries.

In the first five years *Global Health Action* published 336 articles from 919 individual authors across 58 countries. These publications comprised: 87 original research articles, 153 articles within Special Issues or Supplements, 21 editorials, 16 PhD Reviews, and 59 other article types. Many authors were included in more than one publication; the 336 articles included 1,423 individual authors [5].

Other journals have gradually joined the global health open access publishing space. There are similarities and differences in priorities and scope. In *Global Health Action* we focus on the links between science, implementation and action. We continue to make distinctive contributions through *PhD Reviews* as well as *Study Design* and *Capacity Building* papers. We aim to link mentorship to submissions that are not ‘up-to-standard’ but have potential to contribute to global health and action. We strive to narrow the gap between established and emerging researchers. This gap remains starkly reflected in the North-South divide.

**Figure 1.**
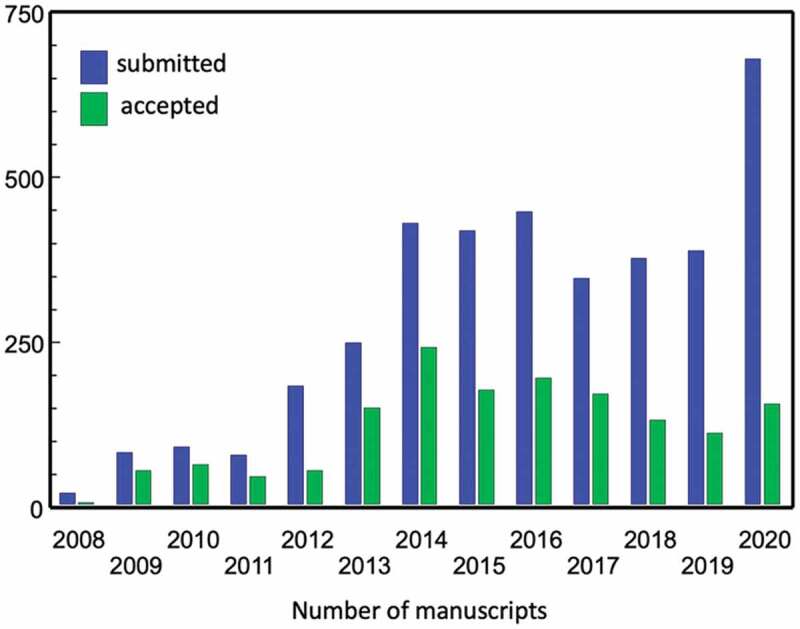
Number of submitted and accepted papers 2008–20

The past ten years has seen a steady increase in submissions to *Global Health Action* (Figure 1). This rose to almost 450 in 2016 and to more than 650 in 2020. Up until 2015, the Journal’s acceptance rate was about 50%. This was partly driven by the higher proportion of invited papers and *Special Issues*. During the last four years only about 30% of submissions were accepted. This decline mirrors a deliberate strategy to further increase the journal´s quality but also a necessary action to cope with the significant increase in submissions within available peer-review resources. Throughout this time *Global Health Action* editors faced the quandary of ensuring quality in the Journal’s publications, without placing unnecessary burdens on external peer reviewers. Hence a new process was proposed.

## Towards efficient editorial and fair peer review work – a validation study

In response to the above issues *Global Health Action* introduced a two-step screening and review process. This replaced the previous system whereby almost all submissions were routinely sent for peer review. Under the new arrangements, all submissions were to be screened in-house for relevance, originality and methodological integrity. At this first step about 50% of submissions are ‘desk-rejected’. Papers which pass move to the second step – external peer review. Typically, about half of the submissions sent for peer review are subsequently rejected, yielding an acceptance rate for all submissions of about 25%.

This two-step process has notable benefits for both authors and reviewers. First, decisions are made in a timelier manner. On average, authors receive notification of the editor’s first decision earlier, regardless of whether their manuscript is desk-rejected or sent for peer review. The process is also more beneficial for reviewers because the manuscripts are generally of a higher quality having passed the initial screening step undertaken by editors.

However, a critical issue is whether the initial screening is sufficiently *fair* to the authors. In order to unpack ‘fairness’, we ask two questions. First, is the process sufficiently *sensitive* in not resulting in too many ‘false positives’, i.e. by not rejecting too many potentially publishable papers? Second, is the process sufficiently *specific* in not resulting in too many ‘false negatives’, i.e. by sending poor quality manuscripts for external peer review? In order to address these questions, we undertook a case study with the objective of validating the Journal’s ‘desk screening’ process.

We invited three experienced academic reviewers to retrospectively ‘post-review’ a sample of manuscripts processed by *Global Health Action* editors during 2020. Their expertise covered many areas of global health including infectious diseases, epidemiology, social medicine, health economics and biostatistics.

Each post-reviewer was given the same sample of 100 manuscripts submitted to *Global Health Action* during 2020. All manuscripts have had a ‘final decision’ outcome. The sample was purposively selected to comprise 50 desk-rejections and 50 papers sent for external peer review. Of the latter, 29 were subsequently published and 21 rejected. The post-reviewers worked independently of one another and were blinded to the final decision outcomes.

The objective of the Journal’s screening step was to identify papers that should not be sent for external review (i.e. desk-rejected). The aim of this study was to ascertain the accuracy of *Global Health Action* editors’ decisions at the initial screening step using the post-reviewers´ recommendations as the ‘gold standard’. We borrowed the statistical measures of performance – *sensitivity* and *specificity* – used in clinical medicine. The *sensitivity* metric is: (true positives)/(true positives + false negatives); the specificity metric is: (true negatives)/(true negatives + false positives).

We defined *high sensitivity* to mean that editors ‘desk-rejected’ a high proportion of submissions at their screening step that a majority (i.e. two or three) of the three post-reviewers also rejected in this study. Conversely, we defined *high specificity* to mean that external peer review was undertaken in a high proportion of the submissions that a majority of post-reviewers recommended for peer review. The ‘positives’ in this context are those papers that were desk-rejected by the post-reviewers. *False positives* are defined as those manuscripts that were desk-rejected by editor*s* but were recommended for peer review by a majority of post-reviewers. *False negatives* are defined as manuscripts sent for peer review by *Global Health Action* editor*s* for which a majority of post-reviewers’ recommended desk-rejection.

Individual agreement for the three post-reviewers was 56%, 64% and 70% which, adjusting for expected agreement, yields Kappa values 0.12, 0.28 and 0.40 respectively (Table 1).

**Table 1.** Agreement between post-reviewers and Global Health Action editors

Post-reviewer	Suggestion by reviewer	Decision by GHA		Total	Agreement	Cohen´s Kappa
		Desk reject	Send for review			
I	Desk reject	21	15	36	56%	0.12
	Send for review	29	35	64		
	Total	50	50	100		
II	Desk reject	38	24	62	64%	0.28
	Send for review	12	26	38		
	Total	50	50	100		
III	Desk reject	40	20	60	70%	0.40
	Send for review	10	30	40		
	Total	50	50	100		

Table 2 shows that, for 72 (45 + 27) of the 100 manuscripts, two or three of the post-reviewers agreed with the Journal’s actual screening decision to either desk reject or send for peer review. For the remaining 28 (8 + 20) manuscripts, two or three of the post-reviewers’ decisions differed from the editors’ decisions. For 16 (4 + 12) of the manuscripts, Journal editors had sent the papers for peer review, but a majority of post-reviewers recommended otherwise (i.e. the false negatives). Out of the 12 (4 + 8) manuscripts that the editors desk-rejected, a majority of post-reviewers recommended peer review (i.e. the false positives).

**Table 2.** Decision outcomes for calculating sensitivity and specificity

Journal initial decision	Three reviewers disagree	Two reviewers disagree	Two reviewers agree	Three reviewers agree	Total
In-house reject	4	8	22	16	50
Send for review	4	12	23	11	50
Total	8	20	45	27	100

From Table 2 we can validate the *Global Health Action’s* in-house screening based on the degree of consistency with all reviewers (= a strong gold standard) giving a *sensitivity* of 80% [16/(16 + 4)] and a *specificity* of 73% [11/(11 + 4)].

When based on the degree of consistency with a majority of post-reviewers (= a medium-strong gold standard) the sensitivity becomes 70% [(16 + 22)/(16 + 22 + 12 + 4)] and the specificity 81% [(11 + 23)/(11 + 23 + 4 + 8)].

**Figure 2.**
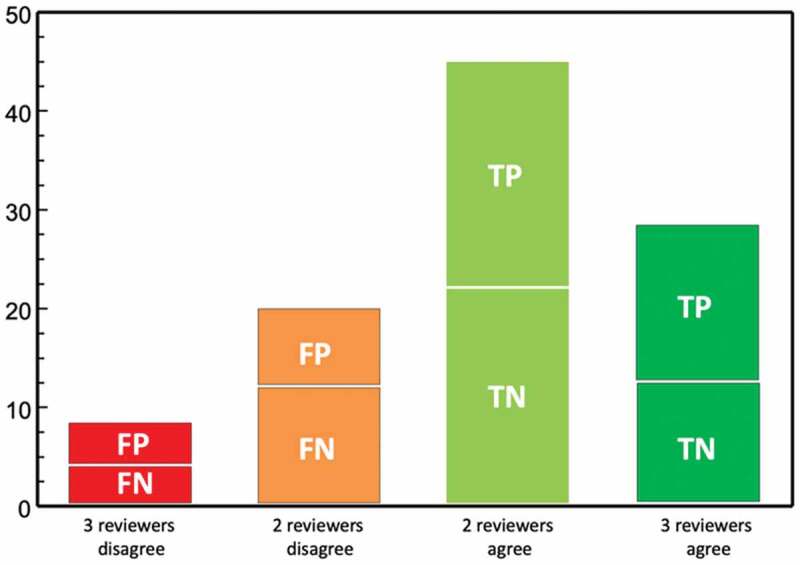
Summary of the validity study in terms of true (TP, TN) and false (FP, FN) positives and negatives

Figure 2 illustrates the location of the 12 *false positives*. These are the 12 manuscripts which *Global Health Action* editors had desk-rejected but for which a majority of the post-reviewers recommended external peer review.

**Figure 3.**
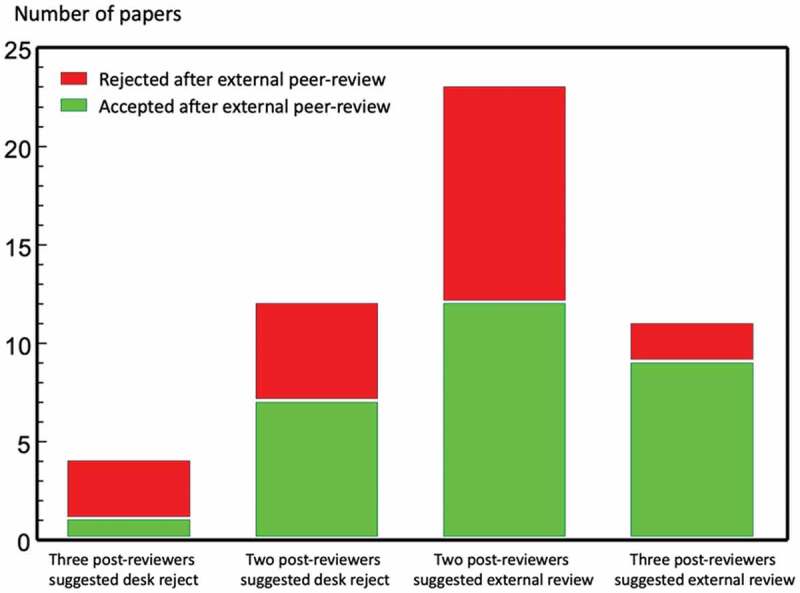
Agreement between post-reviewers´ decisions and final decision outcomes for the 50 papers sent for external peer review

Giving consideration to the outcomes of manuscripts sent for external peer review gives further insights. A closer look at the 16 false negatives i.e. when two or three post-reviewers recommended desk-rejection for papers which editors sent for peer review (Figure 3, first two bars) shows that after external peer review 8 (marked green) were subsequently accepted and 8 (marked red) later rejected. Among the 34 true negatives (last two bars) 21 were accepted (marked green) and 13 were rejected (marked red) after external peer review.

## The way forward

The *Global Health Action* mission is to contribute to bridging the ‘know-do’ gap in global health by disseminating globally relevant information on health from low- and middle-income countries for local action. Our goal is also to support North-South and South-South collaborations involving emerging researchers. The significant rise in submissions in recent years indicates a wide outreach in dissemination and readership. Yet it is also important to critically assess the extent to which goals are being met and shortcomings mitigated.

### The results from the validation case study: do they support GHAs two-stage strategy?

*Global Health Action* strives to have an enabling role vis-á vis emerging authors in low- and middle-income countries. At times this involves ‘lowering the bar’ to give less experienced authors opportunities to have their submissions sent for peer review rather than desk-rejected. The validation study described here aimed to retrospectively assess whether our systematic screening of incoming manuscripts preceding external peer review is *fair, sensitive* and *specific*. The sensitivity and specificity estimates suggest that the Journal’s in-house editorial screening identifies about 70% of sub-standard submissions, and 80% of potentially publishable manuscripts based on the post-reviewers’ assessments. While this is a satisfactory outcome, it is important to recognize the various dimensions and considerations involved in the process of screening.

The issues at stake are illustrated in the four false positive (FP) manuscripts desk-rejected by *Global Health Action* but recommended for external peer review by all three post-reviewers (Table 2). Manuscripts FP-1 and FP-2 used qualitative methods to report on activities by community health workers and nurses in African communities diagnosing and screening for cardiovascular risk factors and diseases. The three post-reviewers noted that both papers were innovative and suitable for external peer review. *Global Health Action* reasons for the desk-rejection included having ‘vague methodology’, being too descriptive and lacking specific methodological details. In retrospect, FP-1 could have possibly been ‘rescued’ through methodological mentorship. However, it would have been questionable as a task for the Journal, as the first author as well as several of the co-authors were affiliated to a well renowned European institute. Low-income country co-authors were neither first nor last authors. Both first and last authors of FP-2 were affiliated to an institute in the USA, while the other co-authors were based with an African site. The first author lacked a higher academic degree.

FP-3 was written in response to the World Health Organization call for monitoring caesarean section data for trends and regional disparities in countries. The three post-reviewers commented that this was policy-relevant for a region in which empirical evidence is scarce, and that the manuscript added to the wider global picture, where caesarean section is both an access and excess issue. *Global Health Action* desk-rejected the manuscript suggesting that it would be better placed in a national or regional journal.

FP-4 was a methodological paper, based on a PhD thesis, describing the development of a program to guide policymakers. The post-reviewers assessed this as an innovative and well-structured approach to program evaluation, that was also highly relevant for global health. *Global Health Action* did not provide a specific reason for the desk-rejection decision.

We next turn our attention to the four false negatives (FNs) that were sent for external peer- review against the recommendations of all three post-reviewers. Three of the four manuscripts were rejected and the fourth was accepted after external peer review.

FN-1 was a cross-sectional survey of community willingness towards health insurance. The three post-reviewers consistently noted that the research question was too ‘descriptive and shallow’ and based on an ‘over-belief’ in numbers. This was consistent with comments by the external peer reviewers who suggested that the concept of ‘willingness’ was undefined and a-theoretical.

FN-2 which was submitted as a *Methods Forum* article, suggested a sub-field of anthropology to pose new questions in global health. Post-reviewers commented that the study was not convincing as a scientific undertaking. The manuscript was rejected after external peer review. The reason for this was that the paper did not convincingly present and discuss how anthropology could practically contribute and influence policy decisions.

FN-3 was a statistical modelling attempt to forecast a country´s life expectancy beyond 2030 using prospective scenarios and potential pathways. Post-reviewers regarded this as being too technical and speculative for *Global Health Action’s* readership, suggesting that the theoretical framework was overly simplistic and built upon an ‘over-belief’ in models. The manuscript was rejected after external peer review because, in the editor’s view, the paper conveyed an obsession with planning.

In summary, the loss of just four out of one hundred manuscripts according to a ‘gold standard’ is a reasonably good record. Out of the eleven externally peer-reviewed manuscripts for which there was ‘gold standard’ agreement with all three post-reviewers (Table 2, Figure 3), nine were accepted for publication in *Global Health Action.*

### What are the lessons for global health action’s further mission?

There are four major criteria on which a decision to either desk-reject or externally peer review is based. These are the *scope* of the study and its relevance for the Journal, whether it adds *new evidence*, whether it is likely that the research question can be answered with the proposed *methodology*, and finally the *readability* of the manuscript. Submissions need not necessarily fall short on readability, but this frequently occurs due to sub-standard language and presentation which distracts from key messages. In such cases sending the manuscript for external peer review is a dis-service to both authors and reviewers. A reasonable decision could be either a desk-reject, or insistence that authors seek professional language editing. In the latter case this is an invitation to authors to resubmit but without commitment that a future submission would necessarily be sent for external peer review.

After a fifteen-year lifespan we ask the question: to what extent has *Global Health Action* disseminated ´results and evidence arising out of the practical implementations of current knowledge’ and published papers ‘suggesting strategies where none exist’ as flagged in our inaugural editorial [2]? The call for filling the ‘know-do’ gaps has certainly been noted by authors in many covering letters. Yet many such submissions have been rejected because authors did not convincingly present the local significance or explain the global implications of their findings. The ‘local-to-global’ relevance therefore needs to be strengthened by the Journal´s author guidelines as well as by the authors themselves. What warrants further emphasis is that, unless a sound reason is given, *Global Health Action* will not accept papers that present or draw down data from low- and middle-income countries where local researchers are not involved as co-authors.

Whether the research question is important and the manuscript adds new knowledge is a matter for peer review, but authors must also clearly articulate the ‘added value’ of their work in order to qualify for a second round of peer review. Our *Paper Context* paragraph introduced a few years back was an attempt to challenge authors to formulate the key highlights from their papers but it is probably not specific enough. A text-box positioned adjacent to the Abstract in the print version may better serve the purpose by clearly stating *Main Findings, Added Knowledge*, and *Global Relevance.*

### Mentorship – a role for an academic journal in the global health field?

Aligned to the Journal’s commitment to both increase the availability of scientific evidence from low- and middle-income countries and contribute to the building of research capacity, we occasionally offer academic *mentorship* to less experienced researchers in order to encourage the development of high-quality manuscripts. We envisage that senior researchers in global health might commit to serve as mentors for *Global Health Action*. The intention is also to improve authors’ capacity to report their results according to the required international standards. This mentorship model has been implemented for some authors of papers within *Special Issues.*

As a case in point, a Special Issue on *Climate Change and Health* was published in due time for the Conference of the Parties (*COP 15*) to the United Nations Framework Convention on Climate Change (UNFCCC) in 2009. We invited two internationally recognized researchers to edit a cluster of submissions on this subject and help recruit researchers to submit manuscripts on the topic [6]. Another example of mentorship can be shown in the supplement *‘Public Health in Vietnam: Here´s the Data, Where´s the Action?’* published in 2013. In this instance three guest editors acted as mentors for local researchers in Vietnam [7].

Would this also be a feasible strategy to apply to individual manuscripts submitted to *Global Health Action*? Could we offer a genuine, fair and non-patronizing approach to the promotion of research capacity in low-income-countries? How could this be implemented in a way that also strengthens South-South ventures? What criteria would apply when selecting candidates for mentorship? Should mentorship only be offered to sole authors lacking institutional support? Should it be an optional request to make during submission? What would be the incentives for mentors or could we count on their altruistic commitment? Many questions about the optimal role of a journal like ours remain and we would welcome the sharing of experiences and views on these issues.

We believe that mentorship in scientific writing for publishing should be offered by journals that claim to make a contribution towards narrowing the digital and information gap in global health. Only by involving local researchers, as stakeholders and collaborators, can we collect valid data from communities in low-income countries. Only through them and their cultural knowledge and competence, can we ask the critical questions. Most importantly, only then can our research endeavors be ethically justified.
